# Nivolumab plus chemotherapy versus chemotherapy alone as first-line treatment for advanced gastric, gastroesophageal junction, and esophageal adenocarcinoma: a cost-effectiveness analysis

**DOI:** 10.1186/s12962-023-00476-2

**Published:** 2023-09-13

**Authors:** Peng-Fei Zhang, Xuan-Qiong Shi, Qiu Li

**Affiliations:** 1 Gastric Cancer Center, Division of Medical Oncology, Cancer Center, West China Hospital, Sichuan University, Chengdu, China; 2 Med-X Center for Informatics, Sichuan University, Chengdu, China; 3 Laboratory of Human Diseases and Immunotherapies, West China Hospital, Sichuan University, Chengdu, China; 4 Institute of Immunology and Inflammation, Frontiers Science Center for Disease-related Molecular Network, West China Hospital, Sichuan University, Chengdu, China; 5 Division of Medical Oncology, Cancer Center, West China Hospital, Sichuan University, Chengdu, China

**Keywords:** Cost-effectiveness, Nivolumab, First-line, Gastric cancer, Markov model

## Abstract

**Background:**

The aim of the study was to evaluate the cost-effectiveness of nivolumab plus chemotherapy as first-line treatment for patients with advanced gastric, gastroesophageal junction (GEJ), or esophageal adenocarcinoma from the perspective of Chinese and US society.

**Methods:**

To conduct the analysis, a state-transitioned Markov model, which included three mutually exclusive health states (progression-free survival (PFS), progressive disease (PD), and death), was developed. Cycle length was set at 3 weeks and lifetime horizon was set at 10 years. Costs, quality-adjusted life years (QALYs), and incremental cost-effectiveness ratio (ICER) were calculated in the analysis. Willingness-to-pay (WTP) thresholds in the model were set at $37,653.00/QALY in China and $100,000.00/QALY in the US, respectively. Meanwhile, one-way sensitivity analyses and probabilistic sensitivity analyses were conducted to investigate the robustness of the model.

**Results:**

Over a lifetime horizon, the ICERs of nivolumab plus chemotherapy versus chemotherapy alone were $430,185.04/QALY and $944,089.78/QALY in China and the US, respectively. Cost of nivolumab and utility for the PFS state had the most significant impact on ICERs both in the US and China based on the results of the one-way sensitivity analyses. In the probabilistic sensitivity analyses, the proportions of nivolumab plus chemotherapy being cost-effective compared with chemotherapy alone were 0%.

**Conclusions:**

In conclusion, nivolumab plus chemotherapy is unlikely to be a cost-effective treatment option compared with chemotherapy alone in the first-line setting of advanced gastric, GEJ, or esophageal adenocarcinoma.

## Introduction

Gastric or gastroesophageal junction (GEJ) cancer is the fifth most common cancer and the fourth leading cause of cancer-related death globally. In 2020, it is estimated that over one million new cases and 769,000 deaths of gastric or GEJ cancer occurred worldwide [1]. Surgery is regarded as the main curative treatment for gastric or GEJ cancer; however, most patients with gastric or GEJ cancer have locally advanced or metastatic disease at the time of diagnosis, and most of patients undergoing gastrectomy will experience disease recurrences [2]. Systemic chemotherapy based on a combination of fluoropyrimidine and platinum is widely used as the first-line therapy for patients with advanced gastric or GEJ cancer, which significantly prolongs overall survival (OS) and improves quality of life (QoL) of these patients [3–5]. In 2010, trastuzumab plus chemotherapy was explored to compare with chemotherapy in first-line setting of patients with advanced or metastatic gastric cancer (GC). Trastuzumab plus chemotherapy significantly prolongs OS of patients with HER2-positive metastatic GC and was approved as the standard first-line treatment for these patients [6]. Despite these progresses, the prognosis of patients with advanced gastric or GEJ cancer remains poor, indicating that novel treatment regimens are urgently needed.

In recent years, cancer immunotherapy, which represents a novel method for cancer treatment, has shown promising antitumor effect in a variety of cancers [7, 8]. Nivolumab is a fully human immunoglobulin G4 monoclonal antibody, which binds to the programmed death 1 receptor (PD-1) and restores T-cell immune activity. In previous study, nivolumab substantially prolonged OS compared with placebo in patients with heavily pre-treated advanced or recurrent GC [9]. Recently, the results of CheckMate 649, which aimed to evaluate nivolumab plus chemotherapy versus chemotherapy alone in first-line setting of advanced gastric, GEJ, or esophageal adenocarcinoma, were reported [10]. Nivolumab plus chemotherapy significantly improved OS and progression-free survival (PFS) compared with chemotherapy alone in patients with a PD-L1 CPS of five or more as well as in patients with a PD-L1 CPS of one or more and all randomly assigned patients, which suggested that nivolumab plus chemotherapy as a promising treatment regimen for patients with advanced GEJ, or esophageal adenocarcinoma.

Regardless of the survival benefits achieved by addition of nivolumab, high cost of nivolumab may counterbalance its antitumor effect and lead to substantial financial implications. Recent years, health expenditure on cancer care has been growing rapidly and has become one of the most severe financial burdens for several countries, especially for countries such as China with limited health resources and large amount of population [11, 12]. To solve the problem, cost-effectiveness analysis is widely used in evaluating the economic implication of treatment regimens [13]. The aim of the study was to evaluate the cost-effectiveness of nivolumab with chemotherapy as first-line treatment for patients with advanced GC from the perspective of Chinese and US society.

## Materials and methods

### Analytic model

**Fig. 1:**
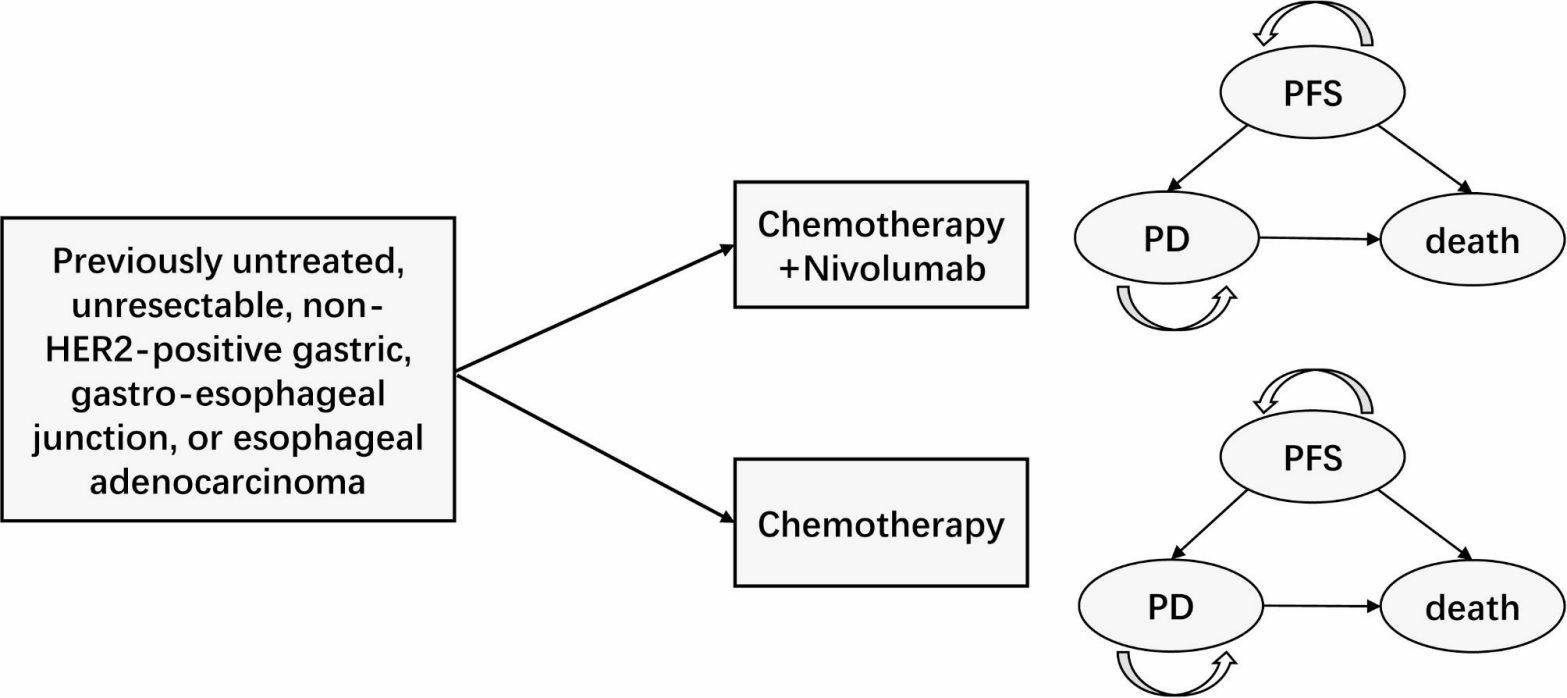
Markov model diagram for patients with advanced gastric, GEJ, or esophageal adenocarcinoma PFS: progression-free survival; PD: progressive disease

A state-transitioned Markov model was developed to evaluate the cost-effectiveness of nivolumab plus chemotherapy compared with chemotherapy alone as first-line treatment for patients with advanced gastric, GEJ, or esophageal adenocarcinoma from a Chinese and US societal perspective (Fig. 1). The model included three mutually exclusive health states (PFS, progressive disease (PD), and death) and integrated efficacy and cost in a hypothetical cohort of patients with advanced gastric, GEJ, or esophageal adenocarcinoma. At the beginning of the model, all patients were assumed to enter the PFS state. Then, these patients can remain in the starting health state or transition to PD or death state at the end of each cycle as described in Fig. 1. Cycle length was set at 3 weeks and lifetime horizon was set at 10 years. Key endpoints of the analysis included costs, quality-adjusted life years (QALYs), and incremental cost-effectiveness ratio (ICER). Willingness-to-pay (WTP) thresholds in the model were set at $37,653.00/QALY (3×per capita GDP of China, 2021) in China and $100,000.00/QALY in the US, respectively. Both costs and health effect were discounted at annual rates of 3%. The model was developed and performed with the Microsoft Excel (Microsoft Corporation, Redmond, WA, USA) and TreeAge software (TreeAge, Williamstown, MA, USA, 2021).

### Patients and treatment regimens

In the Markov model, the hypothetical cohort of patients was simulated based on the baseline characteristics of the patient in the CheckMate 649 study. Hypothetical eligible criteria were: [1] Aged ≥ 18 years; [2] Histologically confirmed previously untreated, unresectable advanced or metastatic gastric, GEJ, or esophageal adenocarcinoma, regardless of PD-L1 expression. Patients were randomly assigned to nivolumab plus chemotherapy or chemotherapy alone group. Nivolumab was administered as following: 360 mg per 3 weeks or 240 mg per 2 weeks. Chemotherapy regimen was based on investigator’s choice (XELOX [capecitabine 1000 mg/m² twice a day, days 1–14 and oxaliplatin 130 mg/m², day 1, every 3 weeks] or FOLFOX [leucovorin 400 mg/m², day 1, fluorouracil 400 mg/m², day 1 and 1200 mg/m², days 1–2, and oxaliplatin 85 mg/m², day 1, every 2 weeks]). Treatment continued until documented disease progression, unacceptable toxicity, withdrawal of consent, or study end. Nivolumab was given for a maximum of 2 years.

### Efficacy, safety, and cost input

**Fig. 2:**
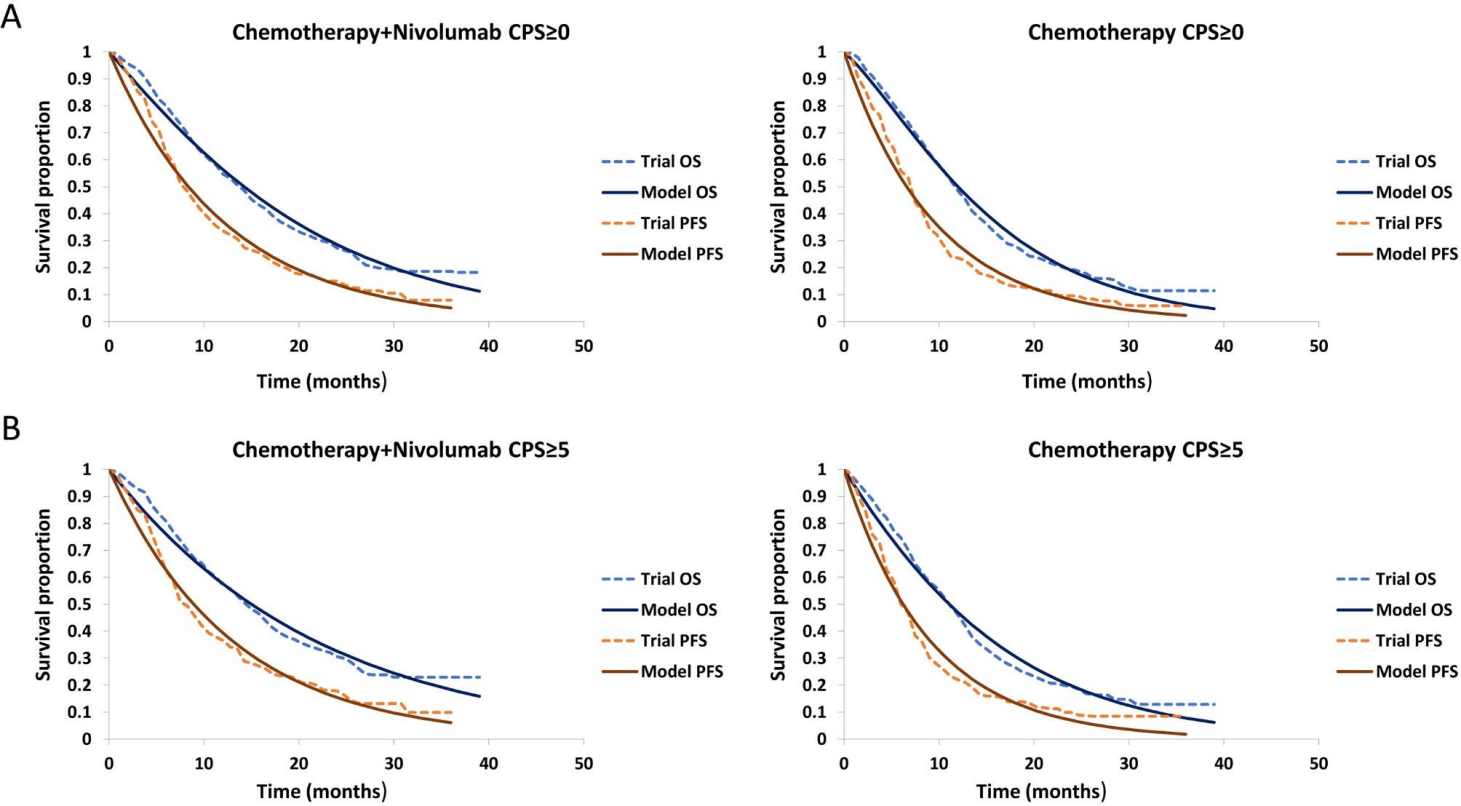
Modelled survival curves for chemotherapy plus nivolumab and chemotherapy alone group PFS: progression-free survival; OS: overall survival; CPS: combined positive score

Transition parameters and probabilities were estimated based on the clinical data from the CheckMate 649 trial. Survival data in each group were extracted from the Kaplan- Meier survival curves using a plot digitizer software (DigitizeIt, version 2.0, www.digitizeit.de) as individual patient data were not available (Fig. 2). In this analysis, grade 3–4 treatment-related adverse events (AEs) with an incidence of ≥ 5% were derived from the CheckMate 649 trial (Table 1). Meanwhile, utility scores for health states, where 1 is full health and 0 is death, was derived from previous literature [14]. The utility scores for each health state were presented in Table 1.

**Table 1:** Key clinical data in the model

Parameters	Nivolumab + chemotherapy	Chemotherapy alone	Reference	Distribution
**Survival data**				
OS (HR), PD-L1 CPS > = 5	0.71 (95% CI:0.59–0.86)	-	[10]	-
PFS (HR), PD-L1 CPS > = 5	0.68 (95% CI: 0.56–0.81)	-	[10]	-
Median OS (months), PD-L1 CPS > = 5	14.4 (95% CI: 13.1–16.2)	11.1 (95% CI: 10.0-12.1)	[10]	-
Median PFS (months), PD-L1 CPS > = 5	7.7 (95% CI: 7.0-9.2)	6.0 (95% CI: 5.6–6.9)	[10]	-
OS (HR), All randomized	0.80 (95% CI:0.68–0.94)	-	[10]	-
PFS (HR), All randomized	0.77 (95% CI: 0.68–0.87)	-	[10]	-
Median OS (months), All randomized	13.8 (95% CI: 12.6–14.6)	11.6 (95% CI: 10.9–12.5)	[10]	-
Median PFS (months), All randomized	7.7 (95% CI: 7.1–8.5)	6.9 (95% CI: 6.6–7.1)	[10]	-
**Grade 3 or 4 AEs, n (%)**				
Nausea	20 (3%)	19 (2%)	[10]	Beta
Diarrhea	35 (4%)	24 (3%)	[10]	Beta
Peripheral neuropathy	31 (4%)	22 (3%)	[10]	Beta
Vomiting	17 (2%)	24 (3%)	[10]	Beta
Fatigue	30 (4%)	16 (2%)	[10]	Beta
Anemia	47 (6%)	21 (3%)	[10]	Beta
Decreased appetite	14 (2%)	13 (2%)	[10]	Beta
Thrombocytopenia	19 (2%)	13 (2%)	[10]	Beta
Platelet count decreased	20 (3%)	19 (2%)	[10]	Beta
Peripheral sensory neuropathy	16 (2%)	14 (2%)	[10]	Beta
Aspartate aminotransferase increased	12 (2%)	5 (1%)	[10]	Beta
White blood cell count decreased	23 (3%)	13 (2%)	[10]	Beta
Alanine aminotransferase increased	6 (1%)	5 (1%)	[10]	Beta
Palmar-plantar erythrodysesthesia syndrome	11 (1%)	6 (1%)	[10]	Beta
Neutrophil count decreased	83 (11%)	67 (8%)	[10]	Beta
Neutropenia	118 (15%)	93 (12%)	[10]	Beta
Asthenia	7 (1%)	10 (1%)	[10]	Beta
Lipase increased	45 (6%)	16 (2%)	[10]	Beta
**Utility (Range)**				
PFS	0.797 (0.638–0.956)	0.797 (0.638–0.956)	[14]	Beta
PD	0.577 (0.462–0.692)	0.577 (0.462–0.692)	[14]	Beta
Death	0	0	[14]	Beta

OS: overall survival; PFS: progression-free survival; AEs: adverse events; PD: progressive disease

Costs of drugs, imaging examination and laboratory tests, AE-related treatments, best-supportive care (BSC), and follow-up were calculated in the analysis. The unit prices of drugs in China were retrieved from the national drug prices or our hospital, while in the US, these data were based on the wholesale acquisition costs from the AnalySource database RED BOOK Online (Table 2). The unit cost of imaging examination and laboratory tests, follow-up, AEs-related treatments and BSC were retrieved from the CMS clinical laboratory fee schedule files and previously published literatures (Table 2) [15–19]. To calculate doses of drugs, we used a mean BSA of 2.1 m^2^ or 1.72 m^2^ for patients in US and China, respectively [19].

**Table 2:** Cost parameters input in the model

Parameters	Value ($)	Range	Resource	Distribution
**Nivolumab (100 mg)**	1342.11 (China)	1073.69-1610.53	Local estimate	Gamma
	3290.22 (US)	2632.18-3948.26	RED BOOK	Gamma
**Nivolumab (40 mg)**	665.40 (China)	532.32-798.48	Local estimate	Gamma
	1316.09 (US)	1052.87-1579.31	RED BOOK	Gamma
**Oxaliplatin (50 mg)**	164.50 (China)	131.60-197.40	Local estimate	Gamma
	98.88(US)	79.10-118.66	RED BOOK	Gamma
**Capecitabine (500 mg)**	1.76 (China)	1.41–2.11	Local estimate	Gamma
	37.71 (US)	30.17–45.25	RED BOOK	Gamma
**5-Fu (250 mg)**	7.61 (China)	6.09–9.13	Local estimate	Gamma
	59.85 (US)	47.88–71.82	RED BOOK	Gamma
**Leucovorin (100 mg)**	2.22 (China)	1.78–2.66	Local estimate	Gamma
	22.8 (US)	18.24–27.36	RED BOOK	Gamma
**Paclitaxel (30 mg)**	59.25 (China)	47.40–71.10	Local estimate	Gamma
	20.16 (US)	16.13–24.19	RED BOOK	Gamma
**Docetaxel (20 mg)**	162.18 (China)	129.74-194.62	Local estimate	Gamma
	211.14 (US)	168.91-253.37	RED BOOK	Gamma
**Carboplatin (50 mg)**	4.40 (China)	3.52–5.28	Local estimate	Gamma
	12.29 (US)	9.83–14.75	RED BOOK	Gamma
**Cisplatin (30 mg)**	2.78 (China)	2.22–3.34	Local estimate	Gamma
	21.20 (US)	16.96–25.44	RED BOOK	Gamma
**Ramucirumab (100 mg)**	-		Local estimate	Gamma
	1427.58 (US)	1142.06-1713.10	RED BOOK	Gamma
**Perbrolizumab (100 mg)**	2596.96 (China)	2077.57-3116.35	Local estimate	Gamma
	5834.45 (US)	4667.56-7001.34	RED BOOK	Gamma
**Toripalimab (2400 mg)**	304. 51 (China)	243.61-365.41	Local estimate	Gamma
	-	-	RED BOOK	Gamma
**Atezolizumab (1200 mg)**	4753.90 (China)	3803.12-5704.68	Local estimate	Gamma
	11032.84 (US)	8826.27-13239.41	RED BOOK	Gamma
**Ipilimumab (50 mg)**	4058.21 (China)	3246.57-4869.85	Local estimate	Gamma
	9273.77 (US)	7419.02-11128.52	RED BOOK	Gamma
**Laboratory tests**	28.99 (China)	23.19–34.79	Local estimate	Gamma
	315 (US)	252–378	[17]	Gamma
**CT**	289.73 (China)	231.78-347.68	Local estimate	Gamma
	231 (US)	184.8-277.2	[17]	Gamma
**Anemia**	508.2 (China)	406.56-609.84	[15]	Gamma
	4368 (US)	3494.4-5241.6	[19]	Gamma
**Neutropenia**	466 (China)	372.8-559.2	[15]	Gamma
	5937 (US)	4749.6-7124.4	[18]	Gamma
**Neutrophil count decreased**	534.4 (China)	427.52-641.28	[16]	Gamma
	5937 (US)	4749.6-7124.4	[18]	Gamma
**Cost of supportive** **care per cycle**	117 (China)	93.6-140.4	[17]	Gamma
	3049 (US)	2439.2-3658.8	[17]	Gamma
**Routine follow-up of patients per unit**	51.5 (China)	41.2–61.8	[17]	Gamma
	422 (US)	337.6-506.4	[19]	Gamma

CT: Computed Tomography

### Sensitivity analysis

A series of one-way sensitivity analyses were conducted to investigate the robustness of the model by varying each parameter to its lower and upper bounds. All parameters were assumed to range between ± 20% and the results of the one-way sensitivity analyses were shown as tornado diagrams. In addition, probabilistic sensitivity analyses were also conducted with each key parameter randomly varied within its distribution range simultaneously for 1,000 iterations.

## Results

### Base case analysis

Table 3 presented the results of the base case analysis. Over a lifetime horizon of 10 years, nivolumab plus chemotherapy group yielded higher effectiveness benefit compared with chemotherapy alone group (1.12 QALYs vs. 0.89 QALYs). The costs of nivolumab plus chemotherapy and chemotherapy alone were $113,897.45 and $14,954.89 in the Chinese societal perspective, while from US societal perspective, the costs of nivolumab plus chemotherapy and chemotherapy alone group were $326,032.70 and $108,892.05, respectively. The ICERs of nivolumab plus chemotherapy versus chemotherapy alone were $430,185.04/QALY and $944,089.78/QALY in China and the US, respectively.

**Table 3:** Base case results of the model

Model outcomes	Nivolumab + chemotherapy	Chemotherapy alone
**US**		
Total costs ($)	326,032.70	108,892.05
Incremental costs	217,140.65	-
Total effectiveness (QALYs)	1.12	0.89
Incremental effectiveness (QALYs)	0.23	-
ICER ($/QALY)	944,089.78	
**China**		
Total costs ($)	113,897.45	14,954.89
Incremental costs	98,942.56	-
Total effectiveness (QALYs)	1.12	0.89
Incremental effectiveness (QALYs)	0.23	-
ICER ($/QALY)	430,185.04	

QALY: quality-adjusted life year; ICER: incremental cost-effectiveness ratio

In addition, we also evaluated the pharmacoeconomic profile of nivolumab plus chemotherapy versus chemotherapy alone in patients with a PD-L1 CPS ≥ 5. Effectiveness benefits were 1.25 QALYs vs. 0.87 QALYs for nivolumab plus chemotherapy group and chemotherapy alone group. In this subgroup, the ICERs of nivolumab plus chemotherapy versus chemotherapy alone were $282,889.68/QALY and $649,647.39/QALY in China and the US, respectively.

## Sensitivity analysis

**Fig. 3:**
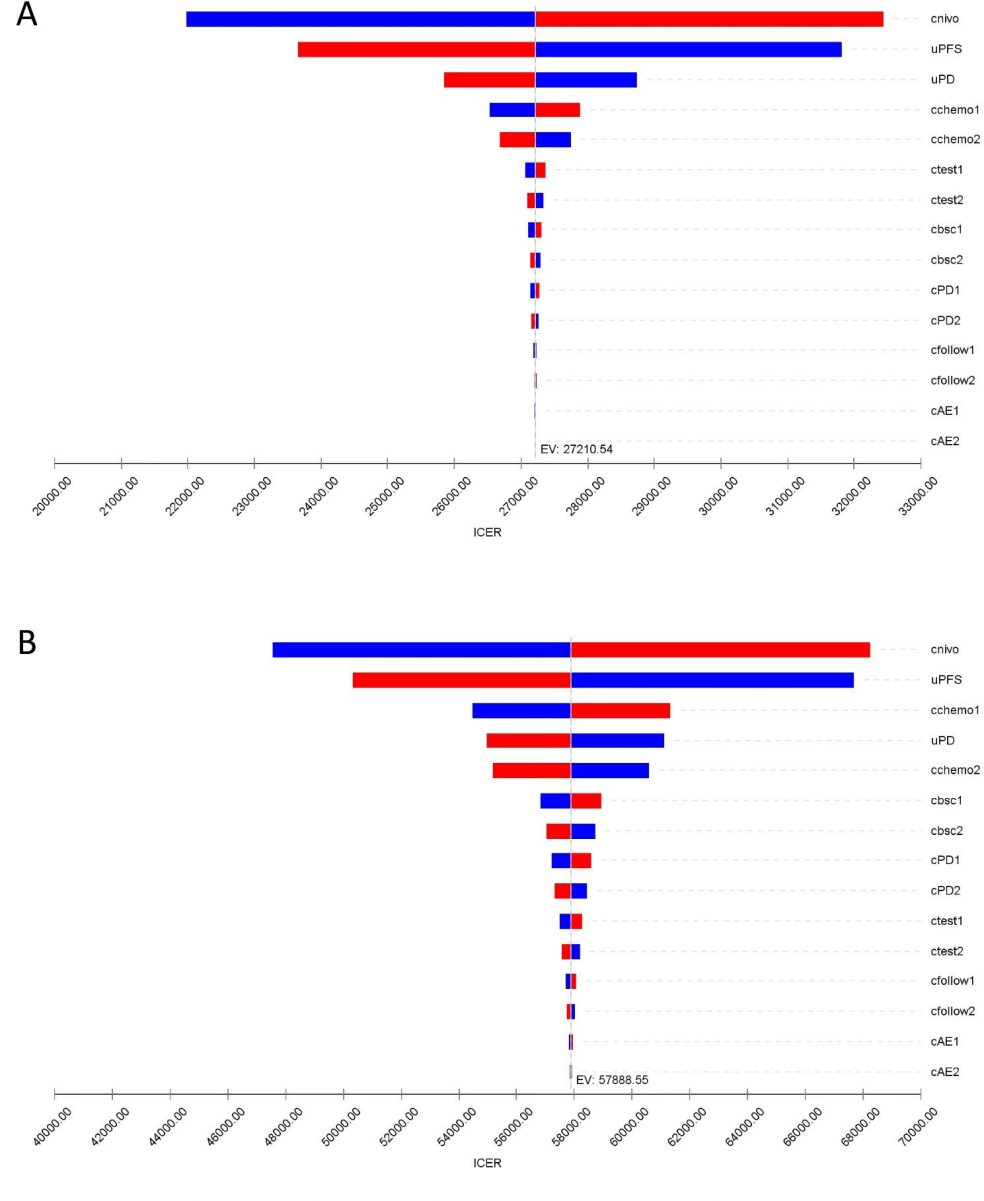
Tornado diagram for one-way sensitivity analyses. **(A)** the Chinese payer’s perspective. **(B)** the US payer’s perspective PD: progressive disease; PFS: progression-free survival; AEs: adverse events; ICER: incremental cost-effectiveness ratio

Cost of nivolumab and utility for the PFS states had the most significant impacts on results of ICERs both in the US and China based on the results of the one-way sensitivity analysis (Fig. 3). Cost of chemotherapy and utility for the PD state had moderate impact on the results. Cost of AE-related treatment, cost of tests, cost of supportive care, and cost of follow-up had little impact on the results of the model. In the probabilistic sensitivity analyses, the proportions of nivolumab plus chemotherapy being cost-effective compared with chemotherapy alone at the WTP thresholds of $100,000.00/QALY in the US and $37,653.00/QALY in China were 0%.

## Discussion

Gastric or GEJ cancer remains one of the most common malignancies worldwide. Patients with advanced gastric cancer have limited treatment options, and the therapeutic effect of current treatment regimens is still not satisfactory. Cancer immunotherapy, which includes immune checkpoint inhibitor, tumor vaccine and adoptive cell therapy, have been widely in a series of cancers and achieved promising antitumor effect. Recently, nivolumab plus chemotherapy have been investigated in the first-line setting of advanced gastric, GEJ, or esophageal adenocarcinoma, which significantly prolonged OS and PFS compared with chemotherapy alone in patients with a PD-L1 CPS of five or more as well as in patients with a PD-L1 CPS of one or more and all randomly assigned patients [10]. However, the price of nivolumab is substantial high. With the widespread use of nivolumab, the dramatic increase in financial burden has become an important issue for doctors, patients and policy makers. Thus, whether its price reflects the drug’s clinical value remain to be determined and an economic evaluation of nivolumab has become urgently needed. In this study, we evaluated the cost-effectiveness of nivolumab plus chemotherapy as first-line treatment for patients with advanced gastric, GEJ, or esophageal adenocarcinoma from the perspective of Chinese and US society. Although nivolumab plus chemotherapy group yielded higher effectiveness benefit compared with chemotherapy alone group (1.12 QALYs vs. 0.89 QALYs), ICERs of nivolumab plus chemotherapy versus chemotherapy alone ($430,185.04/QALY and $944,089.78/QALY in China and the US, respectively) were much higher than the WTP thresholds, suggesting that nivolumab plus chemotherapy is not a cost-effective treatment option compared with chemotherapy alone in the first-line setting of advanced gastric, GEJ, or esophageal adenocarcinoma.

In the one-way sensitivity analyses, the most influencing parameters in the model were cost of nivolumab and utility for the PFS states both in the US and China based on the results of the one-way sensitivity analyses. Recent years, immune checkpoint inhibitors, such as nivolumab and pembrolizumab, have significantly improved survival and quality of life for patients in a series of malignancies. However, not all patients can benefit from the novel treatment, and it is essential to find the most suitable patients with best survival benefits for the immune checkpoint inhibitors. In this analysis, we evaluated the cost-effectiveness of nivolumab plus chemotherapy in patients with a PD-L1 CPS ≥ 5. As expected, ICERs in patients with a PD-L1 CPS ≥ 5 were much lower than those in whole patients. However, these ICERs were also much higher than the WTP thresholds, which suggested that more factors should be considered to select the most suitable patients. In addition, 208 patients enrolled and randomized in CheckMate 649 trial were Chinese. In the subgroup analysis of these patients, nivolumab plus chemotherapy resulted in a more clinically meaningful improvement in median OS (14.3 vs. 10.2 months; HR 0.61 [95% CI: 0.44–0.85]) and median PFS (8.3 vs. 5.6 months; HR 0.57 [95% CI: 0.40–0.80]). Although further exploration may be needed, these results may also influence the pharmacoeconomic profile of combination of chemotherapy and immunotherapy among populations in different regions [20]. On the other hand, the high price of immune checkpoint inhibitors limited their availability, especially in countries with limited healthcare resources. Thus, immune checkpoint inhibitors with low price and high efficacy are urgently needed. Recently, a series of PD-1 inhibitors with lower prices and equal efficacy, such as Toripalimab, Sintilimab, and Camrelizumab were approved in China and was applied to the treatment of a series of cancers, which provides new insights for cancer treatment. Taken esophageal cancer as an example, several studies have demonstrated that pembrolizumab plus chemotherapy is not a cost-effective option for advanced esophageal cancer in the US and China, regardless of PD-L1 expression status [17, 21, 22]. However, sintilimab plus chemotherapy, toripalimab plus chemotherapy and camrelizumab plus chemotherapy were likely to have a cost-effectiveness advantage over chemotherapy alone for previously untreated advanced or metastatic ESCC in China [23–26]. Thus, with the widely application of these drugs, PD-1 inhibitors may become a more cost-effective treatment option in the first-line setting of advanced gastric, GEJ, or esophageal adenocarcinoma.

Several limitations in the analysis should be addressed. First, the cost of grade 1–2 AEs were not included, which may undermine the robustness of the study. Fortunately, the results of the one-way sensitivity analyses demonstrated the economic results were not sensitive to AEs-related parameters. Second, as a trial-based model, the model survival originated from the published data of CheckMate 649 trial. CheckMate 649 trial is a multicenter, randomized, phase III clinical trial comparing nivolumab plus chemotherapy versus chemotherapy alone in advanced gastric, GEJ, or esophageal adenocarcinoma. Although large and well-designed. it might not fully reflect the natural disease course in the real-world. Third, the study merely investigated the cost-effectiveness of nivolumab plus chemotherapy versus chemotherapy alone. Other competing treatment regimens were not included as the absence of head-to-head trials. Fourth, utility scores in the study were derived from previously published literature as the HRQoL data for patients were unavailable in the CheckMate 649 trial, which might lead to bias in the model outcomes.

In conclusion, nivolumab plus chemotherapy is unlikely to be a cost-effective treatment option compared with chemotherapy alone in the first-line setting of advanced gastric, GEJ, or esophageal adenocarcinoma, based on the efficacy reported in the CheckMate 649 study and the current prices of these drugs.

## Data Availability

The data generated in the current study are available from the corresponding author on reasonable request.
